# Hepcidin is elevated in mice injected with *Mycoplasma arthritidis*

**DOI:** 10.1186/1476-9255-6-33

**Published:** 2009-11-24

**Authors:** Curry L Koening, Hong-Hua Mu, Adam Van Schelt, Eric Lo, Diane M Ward, Jerry Kaplan, Ivana De Domenico

**Affiliations:** 1 Division of Rheumatology, Department of Internal Medicine, University of Utah, School of Medicine, 30 North 1900 East, Salt Lake City, 84132, Utah, USA; 2 Department of Pathology, University of Utah, School of Medicine, 30 North 1900 East, Salt Lake City, 84132, Utah, USA; 3 Division of Hematology, Department of Internal Medicine, University of Utah, School of Medicine, 30 North 1900 East, Salt Lake City, 84132, Utah, USA

## Abstract

*Mycoplasma arthritidis *causes arthritis in specific mouse strains. *M. arthritidis *mitogen (MAM), a superantigen produced by *M. arthritidis*, activates T cells by forming a complex between the major histocompatability complex II on antigen presenting cells and the T cell receptor on CD4+ T lymphocytes. The MAM superantigen is also known to interact with Toll-like receptors (TLR) 2 and 4. Hepcidin, an iron regulator protein, is upregulated by TLR4, IL-6, and IL-1. In this study, we evaluated serum hepcidin, transferrin saturation, ferritin, IL-6, IL-1, and hemoglobin levels in *M. arthritidis *injected C3H/HeJ (TLR2^+/+^, TLR4^-/-^) mice and C3H/HeSnJ (TLR2^+/+^, TLR4^+/+^) mice over a 21 day period. C3H/HeJ mice have a defective TLR4 and an inability to produce IL-6. We also measured arthritis severity in these mice and the amount of hepcidin transcripts produced by the liver and spleen. C3H/HeJ mice developed a more severe arthritis than that of C3H/HeSnJ mice. Both mice had an increase in serum hepcidin within three days after infection. Hepcidin levels were greater in C3H/HeJ mice despite a nonfunctioning TLR4 and low serum levels of IL-6. Splenic hepcidin production in C3H/HeJ mice was delayed compared to C3H/HeSnJ mice. Unlike C3H/HeSnJ mice, C3H/HeJ mice did not develop a significant rise in serum IL-6 levels but did develop a significant increase in IL-1β during the first ten days after injection. Both mice had an increase in serum ferritin but a decrease in serum transferrin saturation. In conclusion, serum hepcidin regulation in C3H/HeJ mice does not appear to be solely dependent upon TLR4 or IL-6.

## Background

*Mycoplasma arthritidis *(*M. arthritidis*) is a rodent pathogen that causes arthritis and a toxic shock-like syndrome in C3H mice. *M. arthritidis *injected mice have been used as a mouse model of human inflammatory arthritis for more than 30 years. Much of the disease phenotype and outcomes are influenced by the *M. arthritidis *mitogen (MAM) superantigen produced by the organism. MAM superantigen activates T cells by forming a complex between the major histocompatability complex (MHC) II molecule on antigen presenting cells and the Vβ chain segments of the T cell receptor (TCR) on CD4+T cells [1]. MAM is a unique superantigen in that it also interacts with Toll like receptor (TLR) 4 and 2 found on cells of the innate immune system [2]. C3H/HeJ mice are particularly susceptible to the effects of the MAM superantigen. Compared to C3H/HeSnJ mice, C3H/HeJ mice have a mutant lps^d ^gene that leads to a hypofunctional TLR4 [3]. Macrophages from C3H/HeJ mice upregulate the number of cell surface TLR2 when exposed to the MAM superantigen [2]. Similarly, C3H/HeJ injected mice have a type 1 cytokine profile (IL-2, interferon-γ, and tumor necrosis factor α) compared to inoculated C3H/HeSnJ mice that have a type 2 cytokine profile (IL-4, IL-6, and IL-10) [3].

Inflammation also alters iron metabolism. Hepcidin, an iron regulatory protein, is produced by hepatocytes and macrophages in response to proinflammatory stimuli. Hepcidin binds to and down-regulates ferroportin, the only known cellular iron exporter, found on the plasma membrane of macrophages, hepatocytes, enterocytes, and syncytial trophoblasts [4]. Iron accumulates in cells that lack plasma membrane ferroportin, which leads to lower amounts of circulating iron available for erythropoiesis. Anemia caused by the upregulation of hepcidin in subjects with inflammation is known as the anemia of inflammation (AI). Little is known regarding the regulation of hepcidin in inflammatory states. Investigators have shown TLR4 activation with lipopolysaccharide (LPS) leads to the upregulation of hepcidin [5,6]. Mice injected with *Borrelia burgdorferi *develop severe arthritis and increased serum levels of hepcidin. A primary mediator of this response is the activation of TLR2 by *B. burgdorferi *on bone marrow macrophages of infected mice [7]. Hepcidin transcription is also upregulated by cytokines such as IL-1 [8] and IL-6. IL-6 increases hepcidin expression through activation of the JAK/STAT3 pathway [9-11]. To determine if hepcidin could be expressed independent of TLR4 activation, we measured serum hepcidin levels in C3H/HeJ mice (TLR2^+/+^, TLR4^-/-^) and compared the values to C3H/HeSnJ mice (TLR2^+/+^, TLR4^+/+^) after infection with *M. arthritidis*. We found that hepcidin levels were increased in both mouse strains and hepcidin regulation was independent of TLR4 and IL-6.

## Methods

A total of 36 female mice (10 weeks), 18 C3H/HeJ (TLR2^+/+^, TLR4^-/-^) and 18 C3H/HeSnJ (TLR2^+/+^, TLR4^+/+^), were injected with *M. arthritidis *in accordance with the University of Utah Animal Resource Center as described previously [12]. Mice were followed for a total of 21 days. Mice were evaluated for arthritis and toxicity as described previously immediately after injection and three, seven, ten, fourteen, and twenty-one days after injection [12]. Three mice from each group were sacrificed under anesthesia on the days of arthritis scoring. Blood was collected by cardiac puncture and serum levels of hepcidin, ferritin, and transferrin saturation were measured as described previously [13,14]. Serum IL-6 and IL-1β levels were assayed using mouse IL-1β and IL-6 ELISA Ready-SET-Go according to the manufacture's instructions (eBioscience, San Diego, CA). Hemoglobin (g/dl) values were measured in both strains of mice in the University of Utah Division of Hematology immediately after infection and then three, 10, and 21 days after infection. Livers and spleens were isolated from each mouse and homogenized. Total RNA extraction was performed using RNeasy (Qiagen, Valencia, CA) according to the manufacturer's instructions. Fifty nanograms of mRNA were used for RT-PCR1 Step according to the manufacturer's instructions (Invitrogen, Carlsbad, CA). The primer sequences used for RT-PCR were HAMP (forward) 5'AGAGCTGCAGCCTTTGCAC3', HAMP (reverse) 5'GAAGATGCAGATGGGGAAGT3', and actin (forward) 5'GACGGCCAAGTCATCACTATTG3', actin (reverse) 5'CCACAGGATTCCATACCCAAGA3'.

Results are reported as mean values ± standard deviation (SD).

## Results

**Figure 1:**
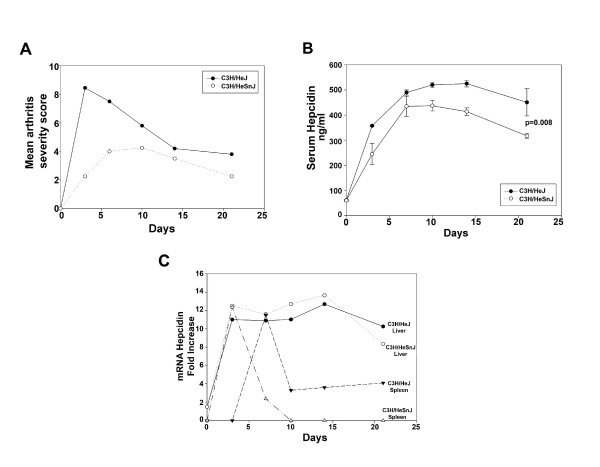
**Serum hepcidin is elevated in mice injected with *M. arthritidis***. **A**. Eighteen female mice (10 weeks) C3H/HeJ and 18 female mice (10 weeks) C3H/HeSnJ mice were injected with *M. arthritidis*, an organism that causes arthritis and a toxic shock like syndrome in susceptible mice. Arthritis severity was scored on days 0, 3, 7, 10, 14, and 21 after injection. **B**. Three mice were sacrificed on days 0, 3, 7, 10, 14, and 21 after injection and serum hepcidin levels were analyzed. Mean values are reported as well as standard error bars. Serum hepcidin levels were significantly higher 21 days after infection in the C3H/HeJ mice than in the C3H/HeSnJ mice. **C**. Hepcidin transcripts were measured from the livers and spleens of each mouse at each time point described in B.

Both C3H/HeJ and C3H/HeSnJ mice developed arthritis within three days of injection. C3H/HeJ mice developed more severe arthritis when compared to C3H/HeSnJ mice. Arthritis severity peaked three days after injection in the C3H/HeJ mice and 10 days after injection in the C3H/HeSnJ mice (Figure 1A). Serum hepcidin increased with arthritis severity in both mouse strains within the first three days of infection. Hepcidin levels reached their highest value 14 days after infection in the C3H/HeJ and 10 days after infection in the C3H/HeSnJ mice. Total serum hepcidin values were higher in the C3H/HeJ mice. The mean hepcidin values for the C3H/HeJ and C3H/HeSnJ mice 21 days after infection were 434.7 ± 54.0 and 311.7 ± 9.5 respectively (Figure 1B). Hepcidin transcripts were detected in the livers of both mouse strains three days after inoculation. Liver hepcidin peaked by day 14 and began to decline by day 21 in both strains. Hepcidin transcripts from the spleens of the C3H/HeSnJ mice were detectable three days after injection but were not detectable in the spleens of C3H/HeJ mice until seven days after injection. Levels quickly dropped in both mouse strains and were undetectable in C3H/HeSnJ mice 10 days after injection. Hepcidin transcripts in the spleens of C3H/HeJ mice decreased by day 10 to levels that were four fold more than C3H/HeSnJ mice (Figure 1C).

**Figure 2:**
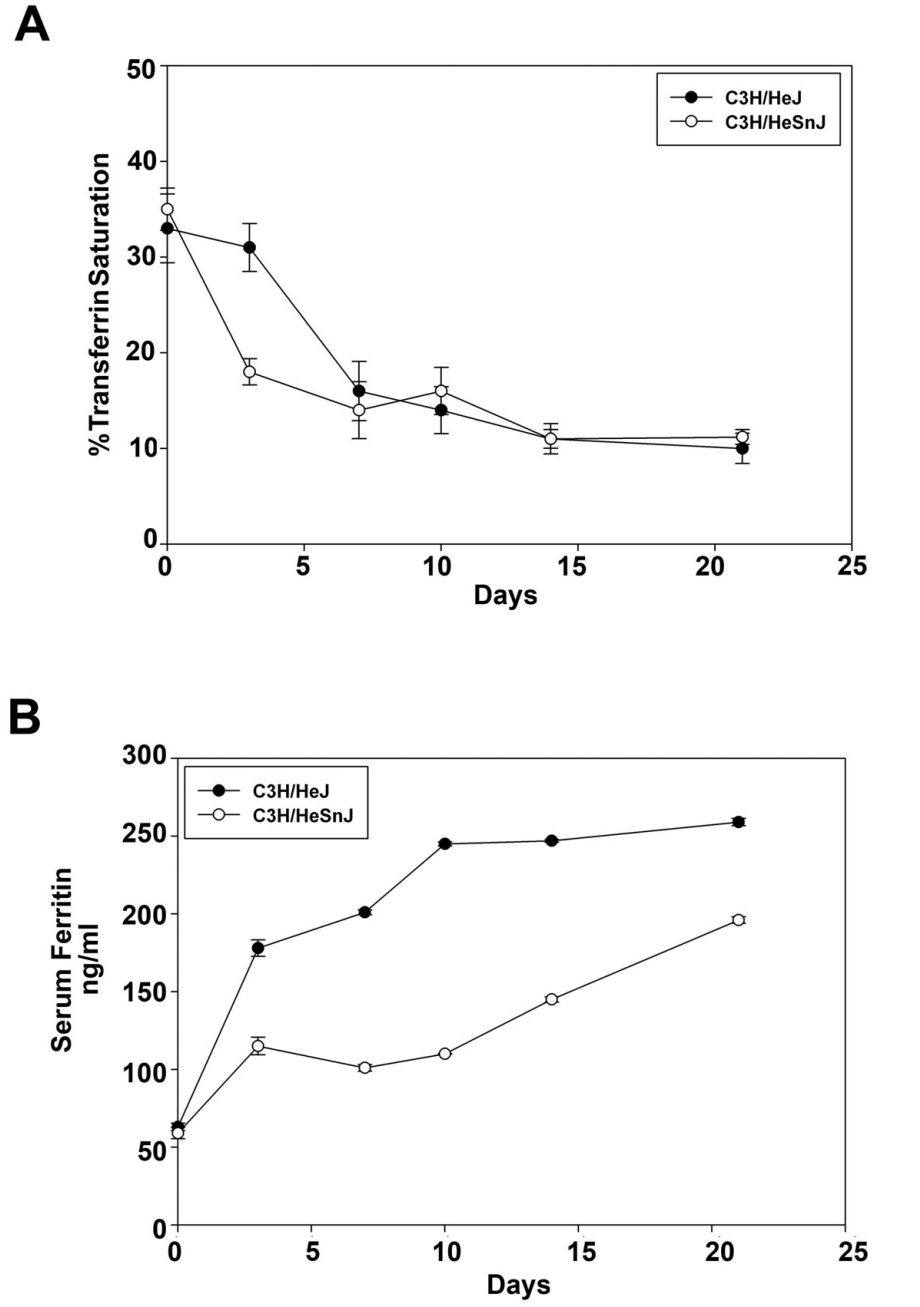
**Mice injected with *M. arthritidis *are hypoferremic**. **A**. Transferrin saturation was analyzed in C3H/HeJ and C3H/HeSnJ mice injected with *M. arthritidis *on days 0, 3, 7, 10, 14, and 21. **B**. Serum ferritin was analyzed at the same time points in A in both strains of mice. Mean values are reported as well as standard error bars.

Transferrin saturation decreased in both groups of mice. Values declined more rapidly in the C3H/HeSnJ mice than in the C3H/HeJ mice but stabilized in both mice seven days after injection and remained similar through the end of the study (Figure 2A). Infected mice from both stains also had lower serum iron levels (data not shown). Serum ferritin values increased in both groups but were higher in C3H/HeJ mice compared to C3H/HeSnJ mice (Figure 2B). Hemoglobin levels in C3H/HeJ mice were lower at the end of the experiment than at the start (Table 1). In contrast, hemoglobin levels in C3H/HeSnJ did not change over the course of the experiment.

**Table 1:** Mean hemoglobin values ± SD were measured in C3H/HeJ and C3H/HeSnJ mice after injection with *M. arthritidis*.

	Day 0	Day 3	Day 10	Day 21
	
C3H/HeJ	14.425 ± 0.424	10.825 ± 7.141	13.200 ± 0.353	10.625 ± 0.565
**C3H/HeSnJ**	10.875 ± 0.919	10.775 ± 2.404	12.275 ± 0.636	10.200 ± 3.670

**Figure 3:**
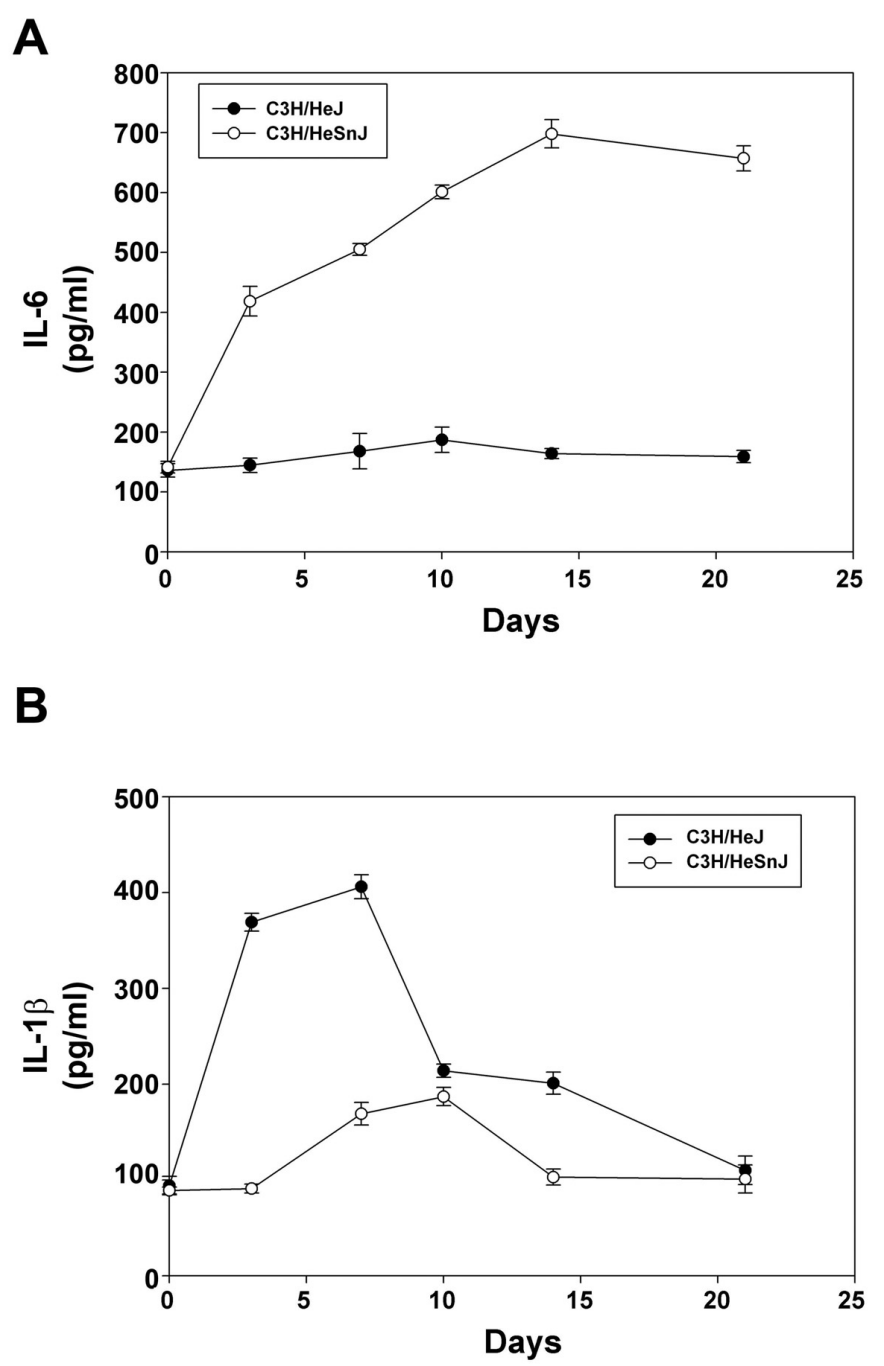
**Serum IL-6 and IL-1β are increased in mice injected with *M. arthritidis***. **A**. Serum IL-6 levels were measured on days 0, 3, 7, 14, and 21 in C3H/HeJ and C3H/HeSnJ mice after injection with *M. arthritidis*. **B**. Serum IL-1β was measured on the same days as in A. Mean values are reported as well as standard error bars.

Serum IL-6 levels were higher in the C3H/HeSnJ mice compared to C3H/HeJ mice. Serum IL-6 levels did not greatly increase in the C3H/HeJ mice over the course of the experiment (Figure 3A). Serum IL-1β levels rapidly increased in C3H/HeJ mice peaking seven days after *M. arthritidis *injection. These levels were much greater than that seen in the C3H/HeSnJ mice whose levels rose more slowly and did not peak until 10 days after injection. The highest serum IL-1β levels observed in C3H/HeSnJ mice were below the peak levels seen in the C3H/HeJ mice (Figure 3B).

## Discussion

Hepcidin is an important iron regulatory protein that when overexpressed may lead to hypoferremia and anemia. Systemic inflammation increases the levels of circulating hepcidin that binds to and degrades the cell membrane receptor ferroportin. By degrading ferroportin, iron can not be secreted into the plasma and hypoferremia develops. Extended periods of low serum iron decreases erythropoiesis and may lead to anemia. The mechanisms that lead to increased hepcidin expression vary among different inflammatory diseases. Hepcidin transcription may occur secondary to activation of TLR [5-7] or through increased expression of IL-6 or IL-1 [8-11]. Our results show that hepcidin secretion is affected by the presence of TLR4 and TLR2. Most notably, the absence of a functioning TLR4 in C3H/HeJ mice allows for unimpeded TLR2 activation in response to the MAM superantigen [2]. Unimpeded TLR2 activation may lead to the overexpression of hepcidin in the infected C3H/HeJ mice. Previous studies indicate that IL-6, a proinflammatory cytokine, is a major inducer of hepcidin transcription. Our studies show that C3H/HeJ mice express low serum levels of IL-6 but have high levels of IL-1β, a cytokine also known to induce hepcidin transcription. Splenic macrophages activated by TLR appear to be important sources of hepcidin. Our results show that mice livers and spleens have different expression patterns of hepcidin mRNA. Hepcidin secretion from the spleens of C3H/HeSnJ mice and from the livers of both C3H/HeSnJ and C3H/HeJ mice occurs shortly after injection of *M. arthritidis*. However, splenic expression of hepcidin transcripts in C3H/HeJ mice is not detectable until several days after injection. The amount of serum hepcidin contributed by the spleen versus the liver is not known but we speculate splenic hepcidin production contributes a great deal to the serum levels of hepcidin seen in our experiments. Furthermore, both the presence and absence of TLR4 affects hepcidin secretion in response to inflammatory agents.

Our results show that C3H/HeJ mice have higher levels of serum hepcidin and ferritin than C3H/HeSnJ mice and that both strains have low transferrin saturation values 21 days after infection. Hemoglobin values were lower through out the experiment in the C3H/HeSnJ mice compared to the C3H/HeJ mice. We speculate the lower hemoglobin values may be specific for this strain but can not rule out that they may be due to an immediate reaction to the infection. The hemoglobin values for the C3H/HeJ mice were lower at the end of the experiment than at the beginning, but this was not seen in the C3H/HeSnJ mice. We suspect 21 days is not enough time to see a significant decline in hemoglobin values and if these mice were followed for a longer period of time, the serum hemoglobin values would decline further. We also speculate that the variation of hemoglobin values in both strains of mice at each time point represents the diverse systemic responses that can be seen in these mice after infection with *M. arthritidis*.

Limitations to our study include its small sample size and short period of followup. Furthermore, the sickest mice were sacrificed at each time point. Selecting the sickest mice for sacrifice gives a false impression that arthritis improves with time and makes it difficult to calculate statistical differences at each time point. It also allows for healthier mice to be analyzed later in the study and may be another reason why hemoglobin values had not declined further in both strains of mice by the end of the study.

## Conclusion

In conclusion, serum hepcidin regulation in states of inflammation appears more complex than originally thought. Serum hepcidin may be upregulated independently of IL-6 and TLR4 activation. Splenic and liver hepcidin regulation is controlled by different mechanisms. TLR2 appears important in the regulation of hepcidin in *M. arthritidis *infected mice, but further work is needed to determine the exact mechanism of hepcidin expression in these mice.

## Competing interests

The authors declare that they have no competing interests.

## Authors' contributions

CLK, IDD, and JK conceived the study; CLK, IDD, and EL performed the experiments; CLK and IDD authored the manuscript; HHM and AVS performed the *M. arthritis *infection; DMW and JK analyzed the data and approved the manuscript. All authors read and approved the final manuscript.
